# Formulation of Transliposomal Nanocarrier Gel Containing Strychnine for the Effective Management of Skin Cancer

**DOI:** 10.3390/gels9100831

**Published:** 2023-10-20

**Authors:** Perwez Alam, Mohd Imran, Dipak Kumar Gupta, Ali Akhtar

**Affiliations:** 1 Department of Pharmacognosy, College of Pharmacy, King Saud University, P.O. Box 2457, Riyadh 11451, Saudi Arabia; aakhtar@ksu.edu.sa; 2 School of Pharmaceutical Education and Research, Jamia Hamdard, New Delhi 110062, India; imranidrisi00786@gmail.com (M.I.); dipak74gupta@gmail.com (D.K.G.)

**Keywords:** strychnine, transliposomes, skin cancer, permeability, dermatokinetics

## Abstract

Strychnine (STCN) has demonstrated an exceptional anticancer effect against various cancers. However, the STCN clinical utility has been hampered by its low water solubility, restricted therapeutic window, short half-life, and significant toxicity. The objective of this investigation was to design and optimize a formulation of strychnine-loaded transliposomes (STCN–TLs) for dermal administration of STCN to treat skin cancer. The formulations of STCN–TL were examined in terms of vesicle size (VS), polydispersity index (PDI), entrapment efficiency (EE), and in vitro delivery. The improved STCN–TL formulation exhibited VS, PDI, EE, and in vitro delivery of 101.5 ± 2.14 nm, 0.218 ± 0.12, 81.74 ± 1.43%, and 85.39 ± 2.33%, respectively. In an ex vivo penetration, the created STCN–TL formulation demonstrated a 2.5-fold increase in permeability compared to the STCN solution. CLSM pictures of skin (rat) revealed that the rhodamine B-loaded transliposome preparation penetrated deeper than the rhodamine B hydroalcoholic mixture. Additionally, rat skin managed with STCN–TL nanogel exhibited a significant increase in C_skin max_ and AUC_0-8_ compared to rat skin treated with traditional STCN gel. The findings demonstrated that the transliposome preparation might be a suitable nanocarrier for the cutaneous distribution of STCN in the amelioration of skin cancer.

## 1. Introduction

Cancer poses a huge global health concern. Skin cancer (SC) begins with aberrant cell proliferation in the epidermis, the outer layer of skin; this is triggered by unrepaired DNA damage that activates mutations and causes the skin cells to proliferate rapidly and form malignant tumors. The four primary types of skin cancer are squamous cell carcinoma, basal cell carcinoma, melanoma, and Merkel cell carcinoma. The ultraviolet radiation from sunlight and the usage of ultraviolet tanning beds are two of the leading causes of skin cancer [1]. SC is a type of malignancy which is widespread worldwide. In some circumstances, the transdermal route is the most appropriate strategy for skin cancer treatment as it can deliver higher drug concentrations directly to the target site [2].

The transdermal route has recently attracted significant attention. Transdermal drug delivery systems (TDDSs) are potentially advantageous since they allow medications to be transported to tumor tissues while preserving common tissues. The medicine is enclosed within nanocarriers. TDDSs lower drug toxicity and improve medication efficacy by addressing various issues, including drug solubilization and instability [3].

Natural products are being extensively recommended for managing a wide variety of disorders. Strychnine (STCN) extracted from nux vomica seeds, a natural resource, was investigated for its anticancer activity. STCN is a terpene indole alkaloid generated from tryptamine and secologanin [4]. It is a member of the Strychnos family of Corynanthe alkaloids. The enzyme strictosidine synthase catalyzes the condensation of tryptamine and secologanin to generate strictosidine [5]. While the enzymes which catalyze the subsequent stages have not been discovered, the steps have been deduced from the isolation of *Strychnos nux vomica* intermediates [6]. The subsequent step involves hydrolysis of the acetal, which opens the ring by removing glucose to yield a reactive aldehyde. The nascent aldehyde is subsequently reacted with a secondary amine to produce geissoschizine, a common precursor for numerous Strychnos family compounds [4]. It is readily soluble in organic solvents but insoluble in water [7]. Orally administered STCN produces gastrointestinal discomfort and systemic damage. Due to STCN’s low water solubility, restricted therapeutic window, short half-life, and severe toxicity, its clinical use has been limited [8]. Creative STCN medication delivery systems are being sought to resolve these issues.

In Chinese medicine, the dried mature *S. nux vomica* L. seed is a frequent therapy for liver cancer [9]. Its therapeutic virtues were first documented in the Compendium of Materia Medica in 1578 A.D. Nux vomica has shown analgesic, antirheumatic, anticancer, and other medicinal benefits in pharmacological trials [2,10]. However, limitations such as a restricted therapeutic window, a short half-life, and high toxicity limit its practical use. Nux vomica contains around 1.5–5% alkaloids, of which STCN accounts for approximately 70% [11].

STCN has been found to strongly suppress the growth of a human hepatoma cell line [12,13]. *S. nux vomica* has revealed cytotoxic activity against multiple myeloma cell lines. The root extract of *S. nux vomica* was screened using the same cell lines and exhibited dose- and time-dependent anticancer activity [14]. Major constituents present in *S. nux vomica* are alkaloidal and effective against HepG2 cell proliferation, whereas brucine alkaloid causes HepG2 cell death via apoptosis through the participation of caspase-3 and cyclooxygenase-2 [13]. The mature, dry seed of the plant Semen Strychni (*S. nux vomica* L.) exhibits analgesic, detumescent, and heat-diffusing properties in the blood. Semen Strychni mainly comprises STCN (C_21_H_22_O_2_N_2_) and brucine (C_23_H_26_O_4_N_2_), which make up from about 2% to 5% of the total alkaloids present. Semen Strychni is also beneficial in reducing the deadly effects of malignant cells. STCN and nitrogen oxide were discovered to have growth-inhibiting and potentially harmful effects on the tumor cell lines K652, HeLa, and HEP-2. These findings suggest that STCN and nitrogen oxide may work by suppressing the protein synthesis of tumor cells rather than having an immediate impact. STCN has a stronger antimutagenic effect, according to genetic toxicology research using the sister chromosome and micronucleus of bone marrow cells in mice. This finding is consistent with an experimental finding using the Semen Strychni alkaloid to suppress tumor cells. Semen Strychni is used to treat several cancers, including esophageal, skin, and gastric cancers. Clinically, a particular curative outcome exists https://patents.google.com/patent/CN1718190A/en accessed on 14 December 2022. Qin et al. developed a model of in situ transplanted liver cancer in nude mice [15]. To minimize the toxicity of STCN while retaining its pharmacological action, pharmaceutical experts have employed a range of techniques, including frying and sand scalding [16], which might be useful due to the maximum temperature conversion of STCN into its N-oxidized derivatives [17]. This approach can reduce toxicity, but it is a complex and laborious operation. Due to the reduced side effects and lack of pain on the application of transdermal drug delivery systems (TDDSs), these have steadily drawn attention as new technology and dosage types have emerged. Therefore, nanocarriers that can cross the skin barrier and enhance the transdermal absorption of medicines have become the subject of intense investigation. Qin et al. [18] created liposomes with the analgesic properties of STCN for transdermal application with reduced toxicity. However, the permeability of traditional liposomes as a locally acting drug reservoir is restricted to the stratum corneum and top layer of the skin with minimal penetration into the deeper skin layers [19]. Conventional liposomes are modified with added surfactants and ethanol to produce flexible liposomes. This modification increases the deformability of lipid membrane vesicles, improving the permeability of medicines. Compared to typical liposomes, ethosomes and transfersomes demonstrated superior drug encapsulation and enhanced skin penetration of STCN [20]. The transliposomes, novel vesicle carriers created by merging liposomes with transfersomes, displayed greater vesicle mobility, improved biocompatibility, and a higher encapsulation rate compared to liposomes or transfersomes [21]. Evidently, transliposomes are the optimal vehicle for TDDS.

The objective of this article was to design and optimize strychnine-loaded transliposomes (STCN-TLs) by altering independent variables viz. Lipoid S100 (LP), cholesterol (CLT), and sodium cholate (SDC) concentrations. Using Box–Behnken design (BBD), we analyzed the impact of the dependent variables VS, EE, and in vitro release (IVR). Additionally, the optimal STCN–TL preparation was assessed for VS, skin permeability and permeability properties, antioxidant activity, and dermatokinetics.

## 2. Result and Discussions

### 2.1. Optimization of STCN–TL by BBD

The BBD program has generated 17 trials to produce three center point formulations. The quadratic model was determined to best match the results of all 17 trials. Lipoid S100 (X1), cholesterol (X2), and sodium cholate (X3) were chosen as the independent responses, whereas the dependent responses were the VS (Y1), EE (Y2), and IVR (Y3). Table 1 displays the R^2^, SD, and % coefficient of variation (% CV) parameters for the three replies.

**Figure 1:**
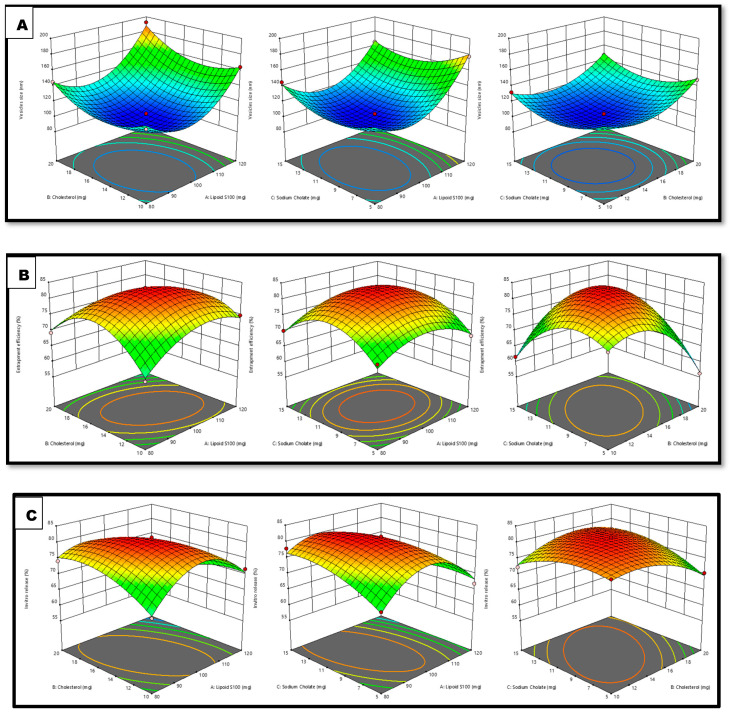
Interpretation of 3D surface plot on the independent variables on (**A**) VS, (**B**) EE, and (**C**) IVR.

**Figure 2:**
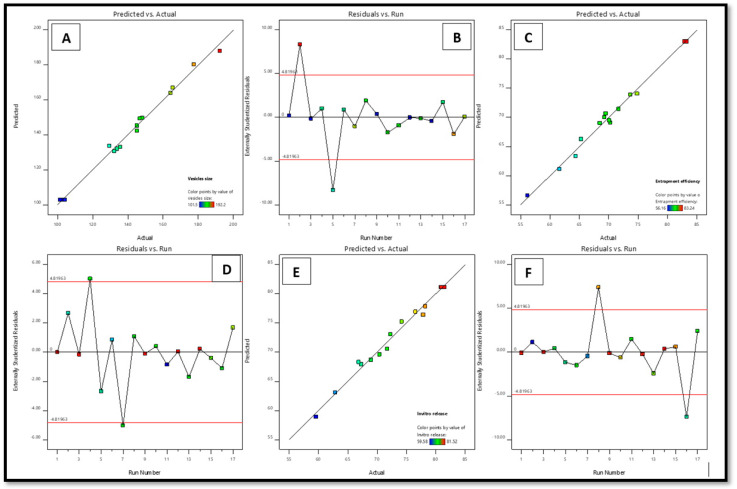
Linear plots between the predicted vs. actual assessments (**A**,**C**,**E**) and subsequent residual plot (**B**,**D**,**F**) for factors vs. and EE and IVR of STCN–TL.

The 3D graph depicts the influence of the specified independent variables on VS, EE, and IVR (Figure 1) and Figure 2 displays the depiction of the linear connection between experimental data and the predicted values derived from the Box–Behnken Design method, along with the associated residual plots for three specific responses.

#### Response 1 (Y1): Variable Impact on vs. Parameter

The practically observed VS throughout all 17 runs was in the range of 101.5–192.2 nm (Table 1).

VS = +102.86 + 18.25 X_1_ + 8.86 X_2_ − 0.7125 X_3_ + 3.10 X_1×2_ − 5.90 X_1_X_3_ + 0.4750 X_2_X_3_ + 34.81 X_1_^2^ + 20.08 X_2_^2^ + 17.78 X_3_^2^

The above equation demonstrates that the Lipoid S100 and cholesterol beneficially impacted the VS. Thus, increases in the quantities of lipid and cholesterol led to an increase in the size of vesicles, since this increases the bilayer’s breadth and accordingly increases the vesicles’ size [21]. The sodium cholate concentration is also crucial for vesicle production. Increasing the sodium cholate concentration decreases the size of STCN–TL vesicles. The size of vesicles typically reduces with an increase in surfactant concentration, carbon chain length, the hydrophilicity of the surfactant head group, and the hydrophilic–lipophilic balance. Elevated surfactant concentration can also result in a rise in charge, subsequently reducing vesicle aggregation and improving system stability [22].

#### Response 2 (Y2): Variable Impact on EE parameter

The practically observed EE throughout all 17 runs was range 56.16–83.24% (Table 1).

EE = +83.02 + 0.2800 X_1_ − 1.74 X_2_ + 0.5263 X_3_ − 3.61 X_1_X_2_ + 0.3050 X_1_X_3_ + 6.90 X_2_X_3_ − 5.40 X_1_^2^ − 9.19 X_2_^2^ − 8.05 X_3_^2^

Lipoid S100 and sodium cholate significantly influenced the EE. A favorable association existed between Lipoid S100 and sodium cholate concentrations and STCN EE in transliposome (TL) vesicles. The EE marginally improves with an increase in sodium cholate quantity within the range of 5 to 15 mg. Similarly, raising the Lipoid S100 concentration from 80–120 mg enhanced the %EE. This is mainly due to the formation of many transliposome vesicles, which increases the domain’s size and creates room for drug entrapment [23].

Cholesterol was incorporated into the preparation to yield stable vesicles; it primarily inhibits outflow, stabilizes the bilayer, and reduces solute absorption into the aqueous cores of the vesicles [24]. Based on the equation, it was determined that cholesterol detrimentally impacted the EE. When the cholesterol quantity was raised from 10–20 mg, the STCN–TL EE decreased. The present findings are consistent with prior studies. More than a particular quantity of cholesterol which might interrupt the bilayer shape of vesicle membranes resulted in medication loss from the vesicle [25].

#### Response 3 (Y3): Independent Variables on IVR

The IVR from all 17 trials varied from 59.58–81.52% (Table 1).

IVR = +81.09 − 3.41 X_1_ − 1.09 X_2_ + 0.6350 X_3_ − 4.75 X_1_X_2_ − 3.23 X_1_X_3_ + 3.02 X_2_X_3_ − 9.07 X_1_^2^ − 3.84 X_2_^2^ − 2.89 X_3_^2^

According to the above equation, sodium cholate beneficially influences the IVR. The in vitro release of STCN increased after the sodium cholate content was raised from 5–15 mg. Conversely, both lipid and cholesterol detrimentally affect the IVR. The drug release marginally reduced with an upsurge in cholesterol quantity within the range of 10–20 mg [1].

**Figure 3:**
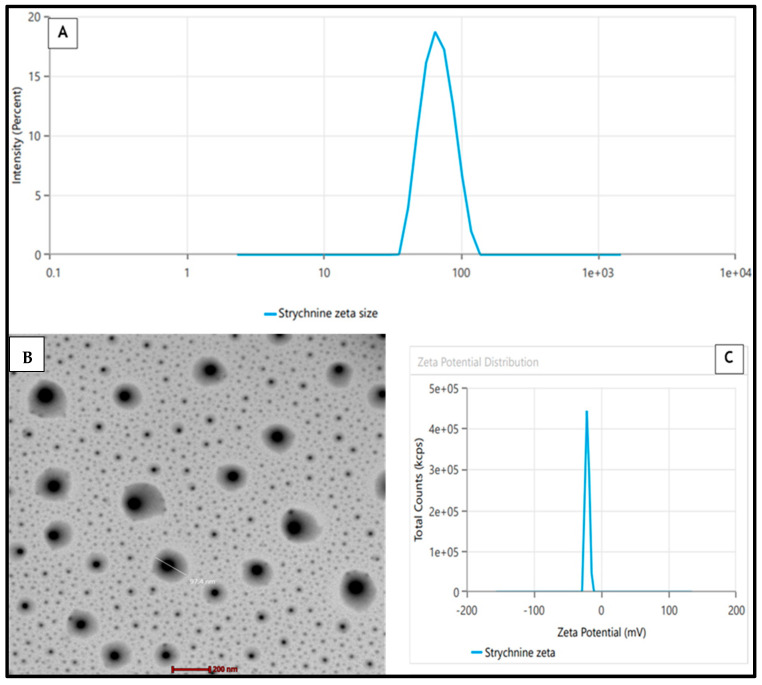
(**A**) Mean vesicle size by zetasizer, (**B**) TEM, (**C**) Zeta Potential of the STCN – TLs preparation.

The BBD software’s (v13) point prediction algorithm was employed to optimize the STCN–TL formulation. Using this method, STCN–TLs with 100 mg of Lipoid S100, 15 mg of cholesterol, and 10 mg of sodium cholate met the requirements for an optimal formulation. The improved STCN–TL formulation exhibited a VS of 101.5 ± 2.14 nm (Figure 3A), a zeta potential of −20.22 mV (Figure 3C), an EE of 81.74 ± 1.43%, and an in vitro release of 85.39 ± 2.31%. The Design-Expert program gave VS (102.86 nm), EE (80.75%), and IVR values close to the projected values (84.68%). Additionally, the improved formulation has a polydispersity index (PDI) of 0.216. In addition to conducting a dermatokinetic investigation, the improved STCN–TL formulation was assessed for VS, IVR, antioxidant activity, pH and texture, skin permeation, and skin penetration depth.

### 2.2. TEM of STCN–TL

A transmission electron microscopy (TEM) representation of the enhanced STCN–TL formulation indicated that the generated vesicle had a well-defined, spherical, and uniformly sized structure (Figure 3B). The VS evaluated by zetasizer equipment employing the dynamic light scattering approach indicated a similar size distribution, as shown in Figure 3A. The TEM image showed no crystalline substance, indicating that STCN was completely entrapped within the vesicular structure.

### 2.3. IVR

**Figure 4:**
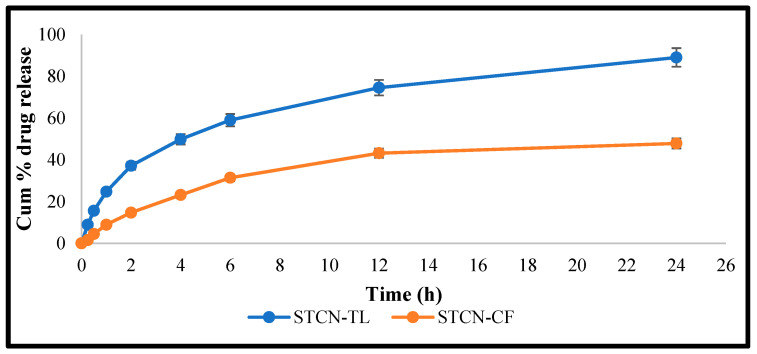
IVR from STCN–TL and STCN suspension at pH 7.4.

The release performance of STCN–TL and STCN conventional preparation (STCN–CF) at maintaining 37 °C under constant agitation at 100 rpm while utilizing the dialysis bag technique revealed a lower percentage of drug release from optimized STCN–CF (40.44 ± 2.16%) than from optimized STCN–TL(89.01 ± 3.71%; Figure 4). Our method of delivering STCN using this TL platform demonstrates a regulated release of the encapsulated drug for up to 24 h, that is substantially comparable to the release model of STCN via liposomal delivery [8,26]. Conversely, the release pattern of STCN from the STCN–CF was substantially low in an aqueous environment (47.86 ± 2.53%) within the experimental design’s limitations, indicating that the created STCN–TL formulation is preferable for delivering STCN. Several models, such as zero-order, first-order, Higuchi, and Korsmeyer–Peppas, were used with the in vitro release research data. The correlation coefficient (R^2^) with the greatest value was favored for determining the release order. The model with the highest correlation coefficient was the Higuchi model (R^2^ = 0.955), followed by the first-order (R^2^ = 0.974) and zero-order (R^2^ = 0.806) models. The optimized STCN–TL had the highest correlation coefficient, indicating that the Higuchi model was the best fit. The release behavior of STCN from STCN–TLs was investigated by setting values in the Korsmeyer–Peppas model; R^2^ was 0.978 and *n* was 0.24, representing that the release of STCN via a STCN–TL follow Fickian diffusion [1].

### 2.4. Antioxidant Activity

**Figure 5:**
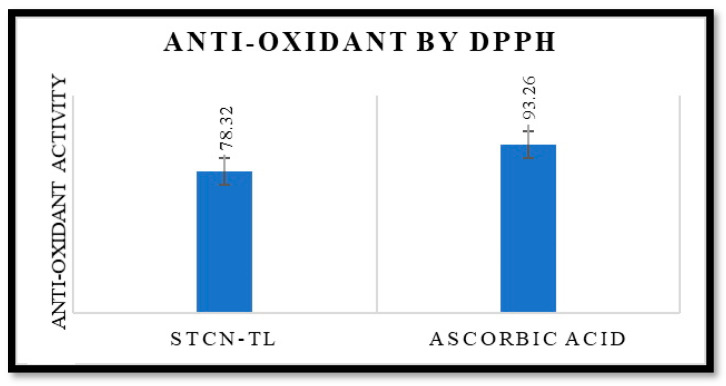
It shows antioxidant activity of STCN–TL and ascorbic acid by DPPH.

Researchers demonstrated the antioxidant activity of this drug by inhibiting the production of free radicals and avoiding lipid peroxidation [27]. The antioxidant potential of STCN is well documented. The antioxidant efficacy of the improved STCN–TL formulation was comparable to that of an ascorbic acid solution. The antioxidant effect of the ascorbic acid solution was 93.26%, whereas that of the STCN–TL optimized formulation was 78.32% (Figure 5). This result validates the antioxidant capability of STCN–TL gel. It was determined that the antioxidant effectiveness of STCN was unaffected by its inclusion in TL gel. Additionally, the preserved antioxidant capacity of STCN will simultaneously enhance the levels of cellular catalase, superoxide dismutase activity, and glutathione peroxidase to inhibit tumor growth [27].

### 2.5. Extrudability, Spreadability, Texture Analysis, and pH of the STCN–TLGel

**Figure 6:**
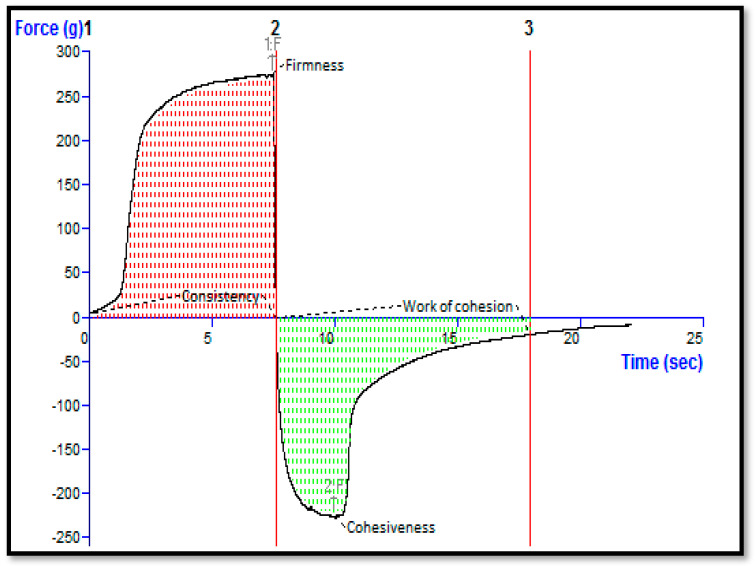
Texture analysis of STCN–TL gel.

Quality control tests demonstrated that gel formulations produced using Carbopol 934 as the gelling agent were superior to those with excellent extrudability and spreadability. The formulation pH for transdermal administration is crucial, especially when the topical distribution is intended for cancer management. Typically, the pH of the cell and extracellular region is close to neutral (pH 7.2); nevertheless, malignant regions are distinguished by a somewhat acidic pH [28]. Thus, the observed pH of 6.2 for the STCN–TL gel formulation was suitable for topical use with skin cancer. The STCN–TL gel had a firmness of 276.13 g, a consistency of 1533.47 g/s, a cohesiveness of −226.09 g, and a viscosity index of −934.89 g/s, as determined by texture analysis (Figure 6). Based on the outcome, it can be extrapolated that the STCN–TL gel preparation has a uniform appearance and consistency since no lumps were observed in it [29].

### 2.6. Stability Testing

An International Council for Harmonization (ICH)-required stability study was performed on the F14 formulation (produced with Carbopol 934), since it exhibited superior quality characteristics, to ensure the quality, safety, and efficacy of the formulation during its shelf-life. After 0, 1, 2, 3, and 6 months of stability testing, no changes were seen in the color, odor, homogeneity, pH, or viscosity of the preparation. According to the findings of the study, the topical gel F2 is highly stable.

### 2.7. Skin Permeation Study

**Figure 7:**
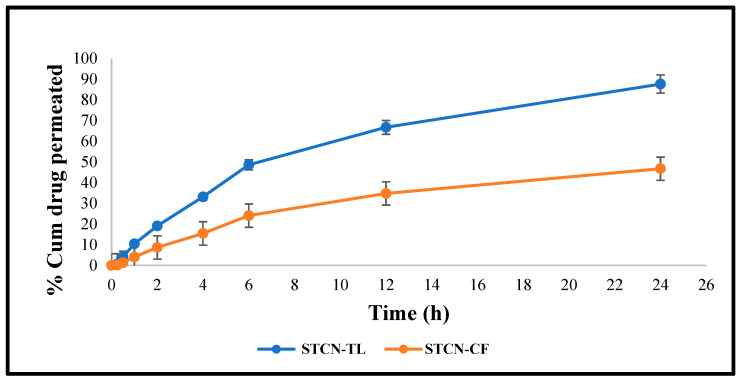
Ex vivo graphs demonstrating the cumulative quantity of STCN entered through skin (rat) by utilizing STCN–TLs gel and STCN–CFs gel.

At 24 h, the cumulative STCN permeation from the STCN–CF was only 46.71 ± 2.16%, in contrast to 87.62 ± 3.02% from the optimized STCN–TL gel (Figure 7). This considerably enhanced permeation into rat skin may be attributed to the compression of TLs by the stratum corneum, where edge activators enhance such permeation. Due to hydrotaxis formation, the difference in hydrostatic force between the skin from two is susceptible to such penetration; this TL permeation is governed by the laws of elasto-mechanics. The flexibility of the vesicle shape reversibly modifies the fluidity of the membrane to assist the movement of vesicle via holes [30].

### 2.8. Inside Skin Permeation Using CLSM

**Figure 8:**
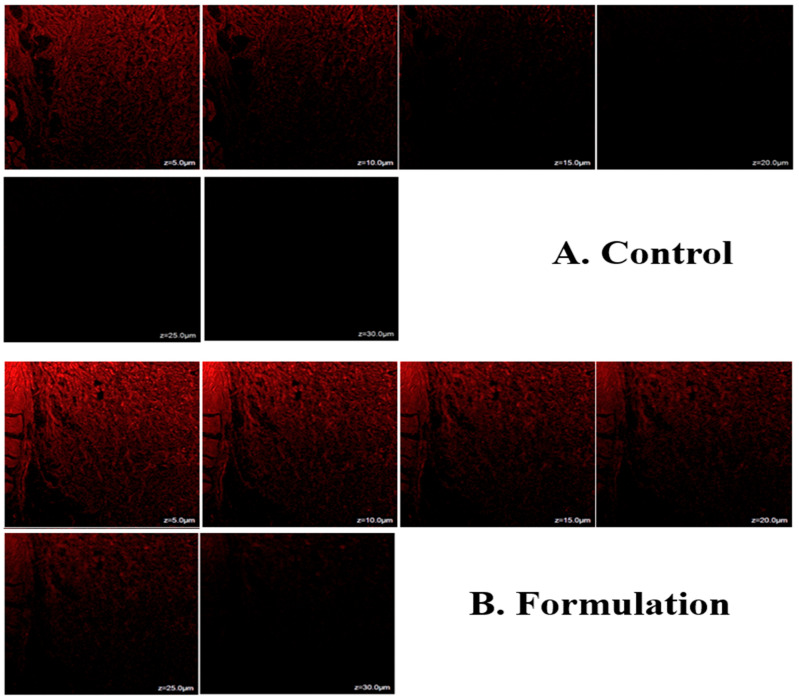
Confocal laser scanning microscopy of control and formulation.

Confocal laser scanning microscopy (CLSM) showed that when rhodamine B was included, the hydroalcoholic solution of rhodamine B remained in the upper skin layers, which only penetrated to a depth of 10 µm, (Figure 8A). STCN–TL gels were well absorbed and showed a depth penetration of up to 30 µm (Figure 8B). The maximum fluorescence intensity was observed in the central region of the skin, indicating that the gel formulation penetrated deeper into the lower epidermis after bypassing the upper layers. In summary, the generated STCN–TL gel transported rhodamine B dye to deeper skin layers. This could also be clarified through the presence of lipid packing defects on animal skin within the subcutaneous layers, which STCN–TL can exploit when penetrating the skin [30]. When applied transdermally, this specifically constructed vesicular lipid particle can transfer the encapsulated medication to the subcutaneous area.

### 2.9. Dermatokinetic Study

**Figure 9:**
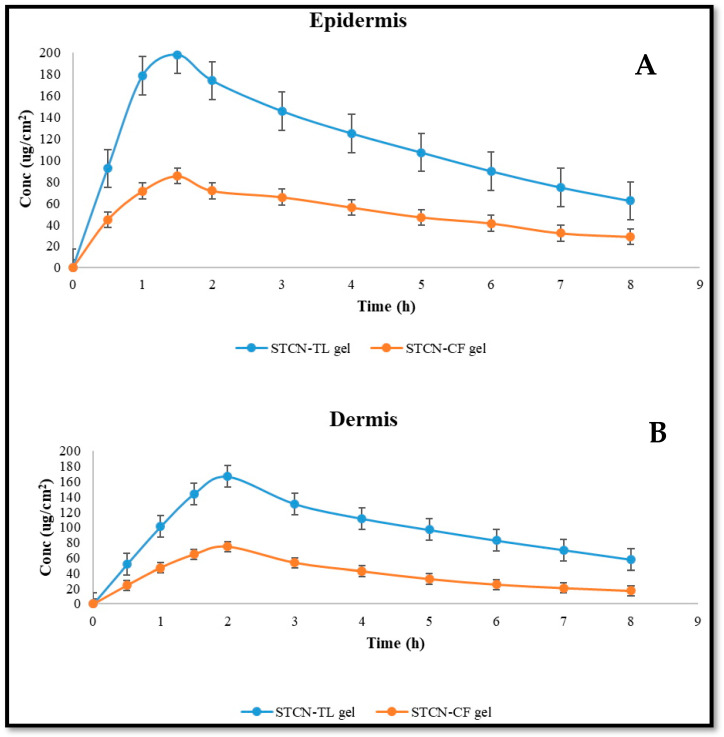
STCN amount in (**A**) Epidermis and (**B**) dermis after topical utilization of STCN-TLs gel and STCN–CFs gel.

The relative concentrations of STCN in the dermis and epidermis of rat skin following management with STCN–CFs and STCN–TLs gel formulations at different time periods are depicted in Figure 9, with the statistical model based on ANOVA with a single component reported in Table 2. Comparing the epidermis and dermis of the rat skin managed with STCN–TLs and STCN–CFs gels revealed that the STCN–TLs gel generated much greater STCN strengths, as determined by C_skin max_ and AUC_0-8_ (Table 2). The utmost retention of STCN–TLs gel may be attributable to the capacity of the vesicles to enhance partitioning by the skin lipid bilayer. In the epidermis, the time to reach maximum level (T_skin max_) of the STCN–TLs gel was comparable to that of the STCN–CFs gel. Based on the results, the STCN strength is detectable 30 min after use on the skin. Compared to the conventional preparation, the STCN–TL administration demonstrated speedy absorption of the medication, with the C_max_ reaching 1.5 and 2 h, respectively, in the dermis and epidermis after topical utilization. The STCN strength dropped with time, up to 8 h into the experiment, at which point the medication concentration was measured.

## 3. Conclusions

In this study, we used BBD software to optimize the STCN–TL formulation. The optimized STCN–TL formulation had a nanoscale VS and showed good EE and IVR. CLSM confirmed that the STCN–TLs gel produced excellent permeability of rhodamine B into the skin (rat) than the rhodamine B blank mixture. Additionally, skin kinetic studies revealed that STCN penetration was better with STCN–TLs gel than with the STCN–CFs gel preparation. The antioxidant effect of STCN was maintained even after being incorporated into the TLs’ vesicle. Typically, the TL vesicle system creates a depot in the deeper layers of the skin and continuously releases the active ingredient over time, which has the benefit of requiring less frequent application. The results indicated that the designed transliposome formulation is a possibly beneficial drug carrier for transdermal administration of STCN to assist in the amelioration in SC.

## 4. Materials and Methods

### 4.1. Materials

Triethanolamine (90279), cholesterol (C8667), and STCN (S0532) were acquired from Sigma Aldrich (Kolkata, India). The supplier of LP was Lipoid GmbH (Ludwigshafen am Rhein, Germany). The SDC was acquired from Thomas Baker (Mumbai, India). We acquired Carbopol 934 and PEG 400 from Merck, (Rahway NJ, USA). Other compounds were acquired from Merck, USA. All solution and compounds used were of analytical quality, and the water used in these experiments was HPLC-grade.

### 4.2. Preparation of STCN–TL Formulation

For the STCN–TL preparation, specific amounts of LP, CLD, SDC, and STCN were combined with chloroform: ethanol (2:1, *v*/*v*) in a suitable flask, followed by 4 h of solvent evaporation in a rotary evaporator. The resultant dried thin film of lipid layer was then rehydrated with a solution of pH 7.4 for 1 h though rotating at 150 revolutions per minute and at room temperature. After, the resultant dispersions were placed in a probe sonicator for 3 min to form tiny vesicles. The TLs were characterized according to their VS, %EE, IVR, and skin permeability. Table 3 displays the chosen independent and dependent responses derived from a Box–Behnken model with three components and three levels.

### 4.3. Optimization of STCN–TL Formulation Using BBD

The three-factor, three-level BBD was used to optimize the formulation of STCN–TL. According to the Design-Expert software (v13), seventeen STCN–TL formulations were obtained and analyzed. Lipoid S100 (X1), cholesterol (X2), and sodium cholate (X3) were chosen as the independent responses, whereas the VS (Y1), EE (Y2), and in vitro release (Y3) were chosen as the dependent variables (Table 3).

### 4.4. VS and PDI

The VS and PDI of STCN–TLs were determined using a zetasizer at 25 ± 1 °C. In this investigation, the preparations were dispersed in a 7.4 pH saline solution and assays were performed in triplicate.

### 4.5. EE (%)

Ultracentrifugation was used to determine the % EE of the TL preparation. The free STCN was separated by centrifuging 2 mL of STCN–TL at 25,000 rpm for 1 h at 4 °C. The gathered supernatant was diluted and filtered, and the quantity of STCN was measured by UV spectroscopy at 265 nm [31]. The EE (percent) was computed using the following formula.

(1) $$\%EE=\frac{\left(Total\;STCN-STCN\;in\;supernatant\right)}{Total\;STCN}\times 100$$

### 4.6. IVR Study

The drug release from STCN–TLs and STCN–CFs was established by utilizing the dialysis bag diffusion method. The improved preparations were placed in dialysis bags, which were then submerged in 50 mL of pH 7.4 phosphate buffered saline at room temperature. At 0, 0.25, 0.5, 1, 2, 4, 6, 12, and 24 h, 1 mL samples were withdrawn and replaced with the same volume of dissolution media. The STCN concentration was measured using a UV spectrometer at 265 nm wavelength [31,32]. Various mathematical models [33] were used to estimate the in vitro release data.

### 4.7. Morphological Analysis of STCN–TLFormulation

A morphological study of the STCN–TL formulation was performed using TEM. A small droplet of the diluted material was applied to a copper grid, allow to dry, and subsequently stained through a 1% PPTA (phosphor-tungstic acid) solution by weight [34,35].

### 4.8. Antioxidant Properties

The antioxidant action of STCN was measured at room temperature using the 2, 2-diphenyl-1-picrylhydrazyl (DPPH) technique [22]. Under ambient conditions, the DPPH free radical solution exhibits a violet color, but when it interacts with an antioxidant, it turns colorless. For the experiment, 0.5 mL of ECL-TL was dissolved in 3 mL of ethanol and combined with 0.3 mL of the ethanolic DPPH solution. Subsequently, this mixture was placed in a dark environment for a duration of 100 min. A spectroscopic analysis at 517 nm was conducted to assess the change in color. The control solution consisted of 3.5 milliliters of 70% ethanol and 0.3 milliliters of the solution (DPPH) [29,36]. The antioxidant activity of the sample, in terms of its ability to scavenge free radicals [37], was quantified as a percentage using the following formula, where “Abs.” represents absorbance [29].

(2) $$\;\%\;antioxidant\;activity=\frac{\left(Abs.\;of\;blank\;-\;Abs.\;of\;the\;sample\right)}{Abs.\;of\;blank}\times 100$$

### 4.9. Preparation of STCN–TL Gel

**Figure 10:**
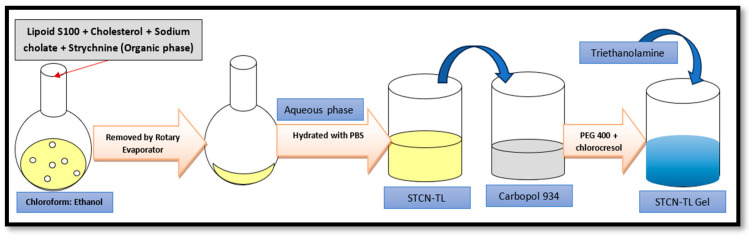
Schematic procedure for gel formulation.

STCN–TL was transformed into a gel formulation; 100 mg of carbopol 934 (1% *w*/*w*) was dissolved in 10 mL of distilled water to create a uniform distribution, and the mixture was left overnight to allow complete swelling of the Carbopol 934. The dispersion was then treated with 15% *w*/*w* PEG 400 and 0.1% chlorocresol (as a preservative), followed by adding tri-ethanolamine as a pH adjuster as shown in Figure 10. Lastly, the tailored STCN–TLs was added drop-wise with steady agitation to these prepared gel bases to generate a homogenous gel formulation [38,39].

### 4.10. Evaluation of pH and the Texture Analysis of the STCN–TL Gel Preparation

The pH of manufactured STCN–TLs gel preparation was determined utilizing a pH meter. The texture of the gel preparation was then assessed by a software-controlled texture analyzer. Texture analyzers are used to measure many properties, such as hardness, brittleness, spreadability, adhesiveness, tensile strength, and extensibility on a vast range of products. Gels can be assessed by measuring their mechanical resistance to stress using texture analysis. In a simple gel strength measurement, 50 gm of the gel preparation (STCN–TLs Carbopol gel) was poured into a 100 mL suitable container to prevent the formation of air bubbles and maintain a flat surface. The gel formulation was then compressed to a depth of 15 mm using an analytical probe at a speed of 2 mm/s in two steps, with a 20-s delay between the completion of the first compression and the start of the second compression. The mechanical parameters of the gels, such as hardness, consistency, cohesion, and viscosity index, were determined by constructing texture analysis curves [22,29].

### 4.11. Extrudability

A clamp was affixed to the crimped end of a closed collapsible tube containing about 20 g of gel to prevent any rollback. Once the cap was removed, the gel was expelled. The amount of extruded gel was collected and weighed. The amount of gel extruded was measured.

### 4.12. Spreadability

Two sets of glass slides of standard size were produced. The enhanced topical gel composition was applied to one of the slides. The second slide was placed on top of the gel, sandwiching it between the two slides for a distance of 7.50 cm; 100 g of gel was placed on the top slides, and the gel was evenly distributed between the two slides to form a thin layer. The weight was removed, and the excess gel was scraped off the slides. The two slides were attached to a platform so that only the top slide could move freely due to the weight connected to it. A 20 g weight was carefully added to the upper slide. Under the influence of the weight, the time necessary for the upper slide to travel 7.50 cm and separate from the lower slide was measured.

### 4.13. Stability Studies of Topical Gel Formulation

The major objective of stability testing is to assess how the drug’s quality changes over time due to changes in temperature and relative humidity. In compliance with ICH guidelines, a six-month stability study of the topical gel formulation was undertaken in a stability chamber. STCN–TLs were placed in a humidity chamber under the following conditions: 25 °C ± 2 °C/60% RH ± 5% RH, 32 °C ± 2 °C/60% RH ± 5% RH, and 40 °C ± 2 °C/75% RH ± 5% RH. Initial, first, second, third, and sixth-month samples were collected and analyzed for color, odor, homogeneity, pH, and viscosity changes.

### 4.14. Skin Permeation Study

The analysis was conducted using Franz diffusion cell (FDC). In this experiment, 1 g of STCN–TL gel was positioned at the upper (donor) portion of the cell non-occlusively, and excised rat skin was used as the membrane. Throughout the experiment, the receiver vehicle, which was pH 7.4 phosphate buffer, was continuously agitated at 600 rpm and maintained at a temperature of 37 °C [40]. At different time intervals (0, 0.25, 0.5, 1, 2, 4, 6 and 12 h), using a sampling tube, approximately 1 mL samples were withdrawn from the receiver part and immediately replaced with 1 mL of a new vehicle [31]. The STCN concentration was measured by HPLC with UV detection at 265 nm.

### 4.15. Estimation of Skin Penetration Depth

The skin penetration depth of the formulations was measured using CLSM. STCN–TLs gel loaded with rhodamine B in a hydroalcoholic mixture was applied uniformly and non-occlusively to the excised rat skin and placed in two various FDC for 8 h at 37 °C. The skin of rats treated with rhodamine B or rhodamine B hydroalcoholic solution loaded with STCN–TL gel was rinsed with distilled water to remove excess STCN–TL gel or hydroalcoholic solution. A microscopic slide was immediately prepared by cutting rat skin into small sections with the stratum corneum facing up and subjected to CLSM. The rat skin was scanned optically using the *z*-axis of the CLSM and 5 µm increments. The optical excitation and measurement of fluorescence emission with an argon laser beam were conducted at 488 and 532 nm, respectively [41].

### 4.16. Dermatokinetic Study

The drug concentrations in different layers of skin after different periods were assessed by spreading the STCN–TL gel on rat skin placed in Franz diffusion cells, as previously reported in the in vitro skin permeation research. However, in this investigation, the entire skin was taken from the FDC after various periods (0, 1, 2, 4, 6, and 8 h) [42]. The skin was rinsed with saline (pH 7.4) and then submerged in hot water at 60 °C for 2–3 min to remove any adhering formulation. The epidermis and dermis layers of the skin sample were separated using forceps. The separated skin layers were sliced into tiny pieces and placed in 5 mL of methanol for 24 h to extract STCN. As stated previously, the resulting methanolic solution was filtered through a membrane, and the STCN content was measured using HPLC [31]. The STCN concentration per cm^2^ of skin was shown against time for the epidermis and dermis separately. T_skin max_, C_skin max_, AUC_0–8_, and Ke (h^−1^) were analyzed as dermatokinetic parameters.

## Figures and Tables

**Table 1:** BBD observed variables of strychnine (STCN)–TL preparation and regression summary analysis results for responses Y_1_, Y_2_, and Y_3_.

Code	Independent Variables			Dependent Variables		
	X_1_	X_2_	X_3_	Y_1_	Y_2_	Y_3_
1	100	15	10	103.5	83.01	81.01
2	120	20	10	192.2	64.35	59.58
3	100	15	10	102.4	82.86	81.11
4	80	15	5	133.7	70.28	68.95
5	80	10	10	129.5	65.28	67.28
6	100	10	15	132.2	61.58	72.27
7	120	15	15	165.4	69.47	62.84
8	80	15	15	145.1	70.06	77.85
9	100	15	10	104.1	82.92	80.98
10	100	20	15	146.8	71.68	76.54
11	100	20	5	148.3	56.16	70.41
12	100	15	10	102.8	83.05	80.84
13	80	20	10	145.1	69.25	74.17
14	100	15	10	101.5	83.24	81.52
15	100	10	5	135.6	73.67	78.21
16	120	15	5	177.6	68.47	66.85
17	120	10	10	164.2	74.82	71.68
Quadratic model	R^2^		Adjusted R^2^	Predicted R^2^	SD	%CV
Response (Y1)	0.9938		0.9858	0.9049	3.34	2.44
Response (Y2)	0.9935		0.9851	0.8966	1.02	1.41
Response (Y3)	0.9877		0.9720	0.8089	1.16	1.57

**Table 2:** Dermatokinetic parameters of STCN–CF gel and STCN–TL gel.

Dermatokinetics Parameters	STCN–CF Gel		STCN–TL Gel	
	Epidermis	Dermis	Epidermis	Dermis
T_skin max_ (h)	1.5 ± 0.2	2 ± 0.2	1.5 ± 0.3	2 ± 0.2
C_skin max_ (µg/cm^2^)	85.15 ± 8.64	74.66 ± 5.21	198.11 ± 3.42	166.75 ± 6.42
AUC_0-8_ (µg/cm^2^ h)	410.49 ± 12.52	307.67 ± 17.65	937.70 ± 27.33	795.01 ± 21.43
Elimination rate constant [Ke (h^−1^)]	0.111 ± 0.052	0.132 ± 0.081	0.117 ± 0.041	0.059 ± 0.023

**Table 3:** BBD independent and dependent responses for the development and optimization of STCN–TL.

Variables	Used Levels		
	Low (−1)	Medium (0)	High (+1)
X1 = lipoid S100 (mg)			
X2 = cholesterol (mg)	10	15	20
X3 = sodium cholate (mg)	5	10	15
X1 = lipoid S100 (mg)	80	100	120
Dependent variables			
Y1 = vesicle size (nm)			
Y2 = entrapment efficiency (%)			
Y3 = in vitro release (%)			

## Data Availability

The data will be available from the authors upon reasonable request.
